# Euglycemic Diabetic Ketoacidosis in Pregnancy: A Case Report and Review of Current Literature

**DOI:** 10.1155/2019/8769714

**Published:** 2019-08-20

**Authors:** Johnny F. Jaber, Matthew Standley, Raju Reddy

**Affiliations:** ^1^Division of Internal Medicine, University of Florida College of Medicine, Gainesville, FL, USA; ^2^Division of Pulmonary, Critical Care and Sleep Medicine, University of Florida College of Medicine, Gainesville, FL, USA

## Abstract

Diabetic ketoacidosis (DKA) in pregnancy is associated with high fetal mortality rates. A small percentage of DKA occurs in the absence of high glucose levels seen in traditional DKA. Prompt recognition and management is crucial. We report a case of a 30-year-old pregnant woman with type 1 diabetes mellitus admitted with euglycemic DKA (blood glucose <200 mg/dL). Initial laboratory testing revealed a severe anion gap acidosis with pH 7.11, anion gap 23, elevated *β*-hydroxybutyric acid of 9.60 mmol/L, and a blood glucose of 183 mg/dL—surprisingly low given her severe acidosis. The ketoacidosis persisted despite high doses of glucose and insulin infusions. Due to nonresolving acidosis, her hospital course was complicated by spontaneous intrauterine fetal demise. Euglycemia and severe acidosis continued to persist until delivery of fetus and placenta occurred. It was observed that the insulin sensitivity dramatically increased after delivery of fetus and placenta leading to rapid correction of ketoacidosis. This case highlights that severe ketonemia can occur despite the absence of severely elevated glucose levels. We discuss the mechanism that leads to this pathophysiologic state and summarize previously published case reports about euglycemic DKA in pregnancy.

## 1. Introduction

Euglycemic diabetic ketoacidosis (EDKA) is a biochemical triad consisting of blood glucose level less than 200 mg/dL, increased anion gap metabolic acidosis, and ketonemia [1]. The incidence of EDKA is reportedly between 0.8% and 1.1% of all pregnant DKA cases [2]. Euglycemia presents a diagnostic challenge often leading providers to believe ketoacidosis is less severe. As a result, EDKA can frequently go unrecognized. Prompt recognition of EDKA is critical in pregnancy, since fetal demise can be as high as 35% without appropriate treatment [3]. We report a case of EDKA in the third trimester of pregnancy and discuss the management challenges in a patient with euglycemia and a high ketone burden. We also summarize other reported cases of EDKA (Table 1).

## 2. Case Presentation

A 30-year-old gravida 2 para 0101 (0 full term, 1 preterm, 0 abortions, and 1 live child) woman at 32 weeks, 3 days gestation presented to our hospital with a two-day history of nausea and vomiting. Her prior medical history included type 1 diabetes mellitus on continuous glucose monitoring and insulin pump. She has had a history of prior episodes of diabetic ketoacidosis requiring hospitalization; however, as her home glucose readings were only marginally elevated at 100–200 mg/dL, she did not think to present to the hospital sooner than she did. Upon initial examination in the emergency department, she appeared uncomfortable with Kussmaul breathing. Fetal heart monitoring showed absent variability and recurrent late decelerations. Her initial admission laboratory results showed a blood glucose of 183 mg/dL, acidosis with an anion gap of 23, pH 7.11, *β*-hydroxybutyric acid (*β*-HA) 9.6 mmol/L (normal 0.02–0.27 mmol/L), and lactate 0.65 mmol/L (normal 0.3–1.5 mmol/L). The patient was admitted to the high risk obstetrics service for further management of her acidosis and resulting fetal distress. As the patient was initially euglycemic, insulin infusion was initiated at 2 units/h as per the institution's DKA protocol. After four liters of bolus intravenous fluids, a maintenance fluid rate of 5% dextrose/0.45% NaCl solution at 250 mL/h was initiated.

**Figure 1:**
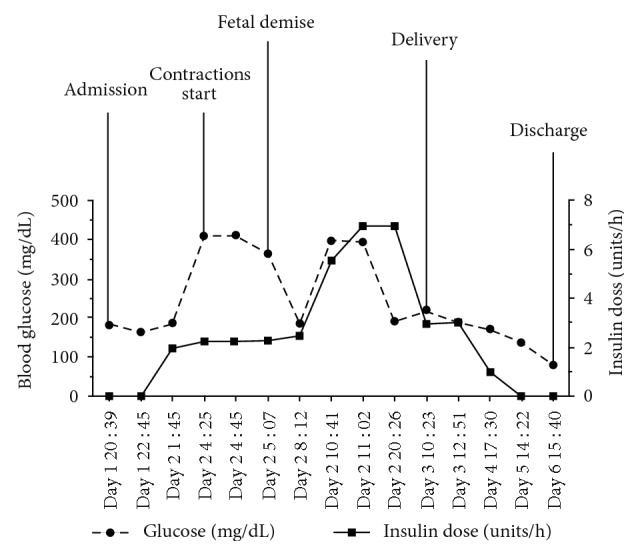
Blood glucose levels and insulin drip rates throughout the patient's hospitalization by day and time of significant events.

Approximately four hours after admission, the patient's euglycemia continued to persist with serum glucose readings averaging 165 mg/dL (Figure 1). Given this euglycemia, the obstetrics team continued to cautiously titrate the patient's insulin drip. Over the next hour, her acidosis progressively worsened with a blood pH nadir of 6.97. Fetal heart tracings continually deteriorated. Unfortunately, the patient was not a surgical candidate for emergent fetal delivery due to her severe acidosis. Bicarbonate containing maintenance fluids were not utilized; however, a total of seven ampules of bicarbonate were administered to the patient throughout her hospitalization to attempt to stabilize the fetus, though bicarbonate containing drips were never initiated. The obstetrics team at this time consulted the medical intensive care unit (MICU) for assistance with further management. At this time, the MICU team consulted endocrinology for assistance with management. The patient's fluids were changed to a 10% dextrose containing fluids at 250 mL/h with the goal of intensifying the insulin infusion to correct the ketoacidosis. Despite this, however, the patient's respiratory status declined to the point of requiring intubation and mechanical ventilation. Approximately eight hours after admission, the fetal heart rate became difficult to detect, and intrauterine fetal demise was declared.

Despite fetal demise, the patient's insulin requirements were still larger than expected at 7 units/h. Endocrinology believed that this was secondary to the placental hormones still in the patient's circulation causing significant peripheral insulin resistance. It was determined that until delivery occurred, the patient would continue to have significant insulin resistance. As soon as one hour after delivery, the patient's insulin sensitivity rapidly improved, and glucose sequestration by the placental circulation disappeared. Acidosis began to rapidly improve. Her glucose now ranged between 200 mg/dL and 400 mg/dL. Her insulin drip was rapidly weaned to 3 units/h, pH continued to rise to 7.33, and the anion gap closed to 7 (Figure 1). The patient was extubated on hospital day 2, and was discharged home three days later without any further events.

## 3. Discussion

EDKA during pregnancy is an obstetric and medical emergency. It is characterized by a state of marked insulin resistance, severe electrolyte derangement, and only marginal elevation of serum glucose. Early recognition and management are crucial, since prolonged ketosis is associated with neurologic complications, and even death in the fetus.

The incidence of DKA is higher in pregnant patients compared to nonpregnant patients, 8.9% vs. 3.1%, respectively [4]. A number of physiologic changes occur in pregnancy predisposing one to severe ketoacidosis [4]. This occurs via multiple mechanisms, the first being marked insulin resistance during pregnancy from several hormones including human placental lactogen, placental insulinase, and progesterone. These hormones peak in the second and third trimesters and can inhibit the effects of maternal insulin resulting in relative insulin deficiency [4]. This could explain why DKA is most common during the second and third trimesters (Table 1). To compensate for the increased insulin resistance, insulin production increases with advancing gestational age [5]. In our case, it is likely that the mother had relative insulin deficiency in the setting of an acute illness and underlying insulin resistance leading to ketosis. Other contributory factors for increased incidence of DKA in pregnancy include lower serum bicarbonate levels (19–20 mEq/L), which occur as a compensatory mechanism for pregnancy-induced respiratory alkalosis.

The mechanism underlying ketosis in pregnancy is similar to that of nonpregnant patients. Ketosis occurs much more rapidly in diabetic patients that are pregnant. In the third trimester alone, the maternal metabolic rate increases by an average of 30% compared to prepregnancy [6]. As a result, even short periods of starvation in pregnant patients predispose them to developing ketosis. Metzger et al. showed that pregnant patients had higher levels of free fatty acids and *β*-HA after a 12 h fast compared to nonpregnant patients [7]. This might serve as a mechanism to provide nutrition to the fetus during periods of decreased caloric intake. In our case, the patient had decreased oral intake for 36 h prior to presentation. The relative insulin deficiency, prolonged starvation, and upregulation of counterregulatory hormones were the likely driving factors for the severe ketonemia observed in our patient.

Not only does ketosis occur much more rapidly in pregnant diabetic patients, it also occurs at much lower serum glucose levels compared to nonpregnant patients [4]. The proposed mechanisms are as follows. First, placental glucose transporters (GLUT-1, GLUT-4, and GLUT-9) are increased during pregnancy. Among those on insulin therapy, placental expression of these receptors is increased even further [8]. As a result of increased placental glucose transporters, maternal levels may be only marginally elevated despite a high ketone burden. Second, euglycemia may also occur due to the physiologic hemodilution that occurs due to increased plasma volume in pregnancy [9]. Third, glomerular filtration rate can increase by 60% from the first trimester to around 4 weeks postpartum, contributing to an osmotic diuresis and thus, absence of marked hyperglycemia despite a high ketone burden [10].

DKA during pregnancy is associated with multiple immediate and late fetal complications. Immediate complications include high fetal mortality rates at 27%–35%, decreased uterine perfusion, fetal hypoxia, and recurrent late decelerations [3,  11,  12]. Our patient had multiple late decelerations on presentation likely reflecting the severity and duration of acidosis. Generally, it is recommended to continue the pregnancy while attempts are made to identify and correct the physiologic derangement. Typically, once the acidosis is corrected, fetal abnormalities improve [4,  12– 16]. Emergent cesarean delivery should only be attempted if the maternal condition worsens, but this is associated with high maternal morbidity and mortality. Long term effects of ketoacidosis include impaired brain development. One study found an inverse relationship with maternal ketonemia and mental development index scores (lower scores indicating inadequate development) at two years of age [17]. Prevention remains a key aspect of managing diabetic pregnant patients. Women should be counseled on checking serum ketones in cases of acute illness or if blood glucose levels begin to rise above their baseline.

## 4. Conclusions

Despite a normal presenting blood glucose level, it is imperative to have a high suspicion for ketoacidosis in an acidotic pregnant patient with diabetes mellitus or gestational diabetes. Placental sequestration of blood glucose can make DKA a diagnostic and therapeutic challenge. Serum ketones must be checked in any diabetic patient during periods of illness. High doses of insulin may be required despite euglycemia to correct acidosis and ketonemia.

Our case highlights the diagnostic and treatment challenges associated with EDKA, and its accompanying complications including fetal demise. The mainstay of treatment remains early recognition and timely administration of fluids, carbohydrates, and insulin.

## Figures and Tables

**Table 1:** Summary of literature describing cases of euglycemic diabetic ketoacidosis in pregnant mothers.

Authors	Age (years)	Diabetic history	Gestational age	Admission blood glucose (mg/dL)	Outcome of mother	Outcome of fetus
Bryant et al. [18]	Describes three cases of diabetic ketoacidosis in pregnant patients with admission glucose <200 mg/dL. No specifics about the three cases are given					
Cardonell et al. [19]	33	Type 2 diabetes mellitus	Third trimester, 35 weeks	134	Discharged home	Emergent C-section at 35 weeks
Chico et al. [14]	29	Type 1 diabetes mellitus	Third trimester, 34 weeks	87	Discharged home	Nonemergent C-section at 34 weeks
Clark et al. [20]	34	Gestational diabetes	Third trimester, 36 weeks	140	Discharged home	Nonemergent C-section at 36 weeks
Cullen et al. [21]	Describes four cases of diabetic ketoacidosis in pregnant patients with admission glucose <200 mg/dL. No specifics about the four cases are given					
Darbhamulla et al. [22]	30	Gestational diabetes	Third trimester, 33 weeks	95	Discharged home	Elective C-section at 39 weeks
Franke et al. [15]	23	Gestational diabetes	Third trimester, 32 weeks	127	Discharged home	Delivery at 38 weeks
Frise et al. [23]	40	Gestational diabetes	Third trimester, 35 weeks	52–85	Discharged home	Emergent C-section at 35 weeks
Guo et al. [4]	29	Unknown	Third trimester, 32 weeks	124	Discharged home	Delivery at 38 weeks
Kamalakannan et al. [24]	28	Type 1 diabetes mellitus	Third trimester, 36 weeks	234	Discharged home	Intrauterine fetal demise
Karpate et al. [25]	25	Unknown	Third trimester, 37 weeks	103	Discharged home	Delivery at 37 weeks
Lucero and Chapela [13]	22	Type 1 diabetes mellitus	First trimester, unknown weeks	153	Discharged home	Unknown
Madaan et al. [26]	30	Type 2 diabetes mellitus	Third trimester, 36 weeks	75–155	Discharged home	Elective C-section at 38 weeks
Madaan et al. [26]	23	Gestational diabetes	Third trimester, 34 weeks	89–164	Discharged home	Emergent C-section at 37 weeks
Montoro et al. [27]	Describes two cases of diabetic ketoacidosis in pregnant patients with admission glucose <200 mg/dL. No specifics about the two cases are given					
Napoli et al. [16]	26	Type 1 diabetes mellitus	Third trimester, 34 weeks	211	Discharged home	Elective C-section at 34 weeks
Oliver et al. [28]	29	Type 1 diabetes mellitus	Third trimester, 28 weeks	245	Discharged home	Elective C-section at 34 weeks
Rivas et al. [29]	39	Gestational diabetes	Third trimester, 32 weeks	120	Discharged home	Emergent C-section at 32 weeks
Tarif and Al Badr [30]	37	Type 2 diabetes mellitus	Third trimester, 35 weeks	77	Discharged home	Unknown
Yu et al. [31]	30	Type 2 diabetes mellitus	Third trimester, 28 weeks	121	Discharged home	Elective C-section at 36 weeks
